# Clinical and Radiological Outcomes Following Medial Pivot Total Knee Arthroplasty: A Retrospective Chart Review Study

**DOI:** 10.7759/cureus.16447

**Published:** 2021-07-17

**Authors:** Wazzan Al Juhani, Mohammed S Alwhaid, Alhanouf M Almuqbel, Alanoud A Alshathri, Salem D Almatrafi, Mohammed Alsalman, Husam Altahan

**Affiliations:** 1 Department of Surgery, Ministry of National Guard Health Affairs, Riyadh, SAU; 2 Orthopaedics, King Abdullah International Medical Research Center, Riyadh, SAU; 3 College of Medicine, King Saud Bin Abdulaziz University for Health Sciences, Riyadh, SAU; 4 Radiation Oncology Section, Oncology Centre, King Faisal Specialist Hospital and Research Centre, Riyadh, SAU; 5 Orthopaedics, Qassim University, Buraydah, SAU; 6 Orthopaedics, AlMaarefa University, Riyadh, SAU; 7 Radiology, King Abdulaziz Medical City, Riyadh, SAU; 8 Orthopaedics, King Abdulaziz Medical City, Riyadh, SAU

**Keywords:** total knee arthroplasty, medial pivot, osteoarthritis

## Abstract

Medial pivot total knee arthroplasty (MP-TKA) is a relatively new design that simulates normal knee mechanics with the aim of enhancing postoperative recovery. Furthermore, it reduces postoperative complications in patients with end-stage osteoarthritis of the knee. No study has been done regarding this topic in Saudi Arabia yet, so we aimed to study the post-operative clinical and radiological outcomes of MP-TKA, as well as the postoperative complications. A retrospective cohort chart review study was conducted on 46 patients and 70 knees after applying our inclusion/exclusion criteria. The patients were followed up for an average period of two years. Clinical outcomes were assessed pre- and postoperatively by the validated Saudi Arabian version of the Knee Injury and Osteoarthritis Outcome Score (KOOS), as well as radiological outcomes and postoperative complications gathered from patients’ charts. The postoperative KOOS score showed a statistically significant improvement in pain, symptoms, and activities of daily living in comparison with the preoperative score (P-value < 0.0001). The mean time until ambulation and length of hospital stay were five and 14 days, respectively. Four patients (8.7%) showed radiological complications. Deep vein thrombosis was observed in only two knees (4.3%), and there were no revision cases. Thus, MP-TKA has been shown to improve pain, symptoms, and activities of daily living with a relatively short time until ambulation and length of hospital stay, in addition to a low incidence of postoperative and radiological complications.

## Introduction

Total knee arthroplasty (TKA) is the most practical and well-established procedure for treating end-stage knee disease, restoring knee function, and relieving pain. Osteoarthritis (OA) is the most common indication for this procedure, with 87% of TKAs being performed on OA patients in the United States in 2016 [1-4]. When effective treatment other than TKA is unavailable, a trial of nonsurgical interventions including orthosis, physical therapy, lifestyle modification, and pharmacological management is done in a stepwise manner until the point of severe pain and limited range of motion [4-6]. The satisfaction rate reported after TKA is approximately 80%, meaning one in five patients who are not completely satisfied [7]. Many factors contribute to postoperative satisfaction, such as meeting preoperative expectations, particularly in relatively young patients who normally have an active lifestyle and perform sports activities [8].

Multiple studies have been done using both fluoroscopic and magnetic resonance imaging to understand normal knee kinematics. Such studies have shown that during knee flexion, the lateral femoral condyle slides on the lateral tibial plateau while the medial femoral condyle and tibial plateau elicit a pivoting movement with a stable contact point between them [9]. In other words, the medial knee compartment works like a ball-and-socket joint, while the lateral femoral condyle translates in an anteroposterior fashion and rotates around the medial compartment during flexion [10]. Furthermore, the medial tibial plateau has a concave shape, while the lateral plateau is more convex, and 60% of the weight is transferred to the medial knee side. In consideration of all of these findings, the concept of medial pivot knee kinematic has emerged [3]. 

Over the years, the performance of TKA has increased, and consequently, the revision rates have as well [11]. Until recently, studies have shown that neither implant designs nor posterior-cruciate retention nor replacement is able to simulate optimal knee kinematics, along with the inability to achieve the anticipated movement and full range of motion. These factors are both important aspects that allow for the improvement of function and the restoration of quality of life [1,4]. This is attributed to what is described in the literature as the paradoxical motion, which is an alteration in the normal knee kinematics causing abnormal anterior sliding of the femoral component on the tibial plateau. This is in addition to not simulating normal medial pivot kinematics of the knee, as described above [3]. 

**Figure 1:**
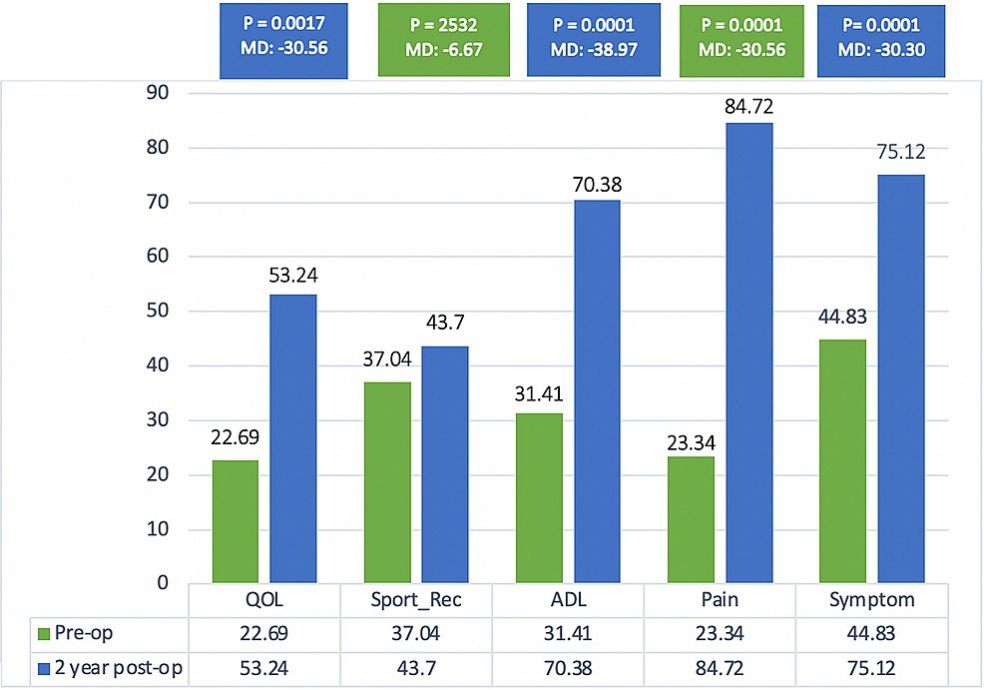
Knee Osteoarthritis Outcome Score (KOOS). ADL: activities of daily living; Sprt_Rec: sports and recreational activities; QoL: quality of life.

In 1998, the medial pivot TKA (MP-TKA) design emerged to simulate normal knee kinematics, improve steadiness, and reduce the unwanted outcomes seen with traditional TKA designs, such as patellar shift, unrecovered mechanics, and limited range of motion [3-4,6,10-12]. The medial tibial-femoral contact zone in this design is wider to achieve a balanced distribution of loads, which reduces the risk of polyethylene damage and prolongs the implant’s survival [9,13]. Furthermore, the medial compartment of the tibial insert is deeper and highly conforming with an anterior lip that stabilizes the knee during full range of motion. The lateral tibial insert is less congruent. This configuration grants more freedom during the anteroposterior movement of the lateral condyle (EVOLUTION, Microport Orthopedics, USA) (Figure 1) [10]. Thus, anteroposterior stability can be achieved with low contact stress and a large contact area, which improves the lifetime of the components. The mid- to long-term outcomes following medial pivot TKA are satisfactory, with revision rates ranging from 3.4% to 5.2% at the 10-year follow-up [14].

Another study evaluated the long-term clinical outcomes of medial pivot design and showed a statistically significant improvement in scores on the Knee Society Clinical System (KSS), Western Ontario and McMaster Universities Osteoarthritis Index questionnaire, Short Form (SF-12) questionnaire, and Oxford knee scores. Furthermore, the majority of patients were able to perform age-appropriate activities with knee flexion reaching up to 135°, in addition to reporting a survivorship of more than 17 years [4]. Another study also showed improved clinical outcomes in terms of KSS, functional scores, and postoperative range of motion with survival rates of 98.6% and 99.1% at the five and 10-year follow-up, respectively, without revision rates [13,14]. Moreover, a study compared a medial pivot design with a flat-surface knee design in posterior cruciate-retaining TKA and showed that the medial pivot design involved less surgical time, estimated blood loss, and post-operative pain while achieving better postoperative flexion angles [15].

Since 1998, few studies have investigated the postoperative course following MP-TKA, although most of them have reported assuring and positive feedback [11,14,16,17]. Some studies have reported that the confined medial compartment can lead to excessive insert wear or stress, which consequently lead to failure, whereas others have shown no difference between medial pivot and other TKA designs [11,18-21]. To our knowledge, evidence regarding medial pivot TKA is lacking in the context of Saudi Arabia. Thus, the aim of this study is to evaluate the clinical and radiological aspects in addition to postoperative complications of patients who underwent medial pivot TKA.

## Materials and methods

Study design

A retrospective chart review study was performed using a convenience sampling technique. The study included all patients who underwent MP-TKA from February 2014 to February 2018 at King Abdulaziz Medical City in Riyadh, Saudi Arabia. All of the patients’ operations were performed by the same surgeon. The primary outcomes consisted of the KOOS score from before the operation and average from two years post-operation, while the secondary outcomes consisted of the short and long-term post-operative clinical and radiological outcomes.

Study setting and population

The study was conducted at King Abdulaziz Medical City in Riyadh, Saudi Arabia. This facility contains more than 1,500 beds, and three to four knee replacements are conducted weekly on average. A total of 46 patients (70 knees) were included after applying our inclusion and exclusion criteria.

Inclusion Criteria

All patients who underwent MP-TKA due to OA or for post-traumatic reconstruction from 2014 to 2018 in King Abdulaziz Medical City.

Exclusion Criteria

- Any patient who was lost to follow up.

- Patients with undocumented or deficient post-operative data. 

- Patients with severely deformed knees. 

Our standard protocol for the prevention of thromboembolism was applied for all our patients. The protocol consists of anticoagulant prophylaxis, a pneumatic compression stockings until ambulation, and early ambulation instructions. In addition, all patients received 1 to 2 g of cefazolin at 30-60 minutes before the surgery, depending on their weight. This was continued for one day postoperatively and was given every eight hours to prevent infection. 

Data collection methods

Data were collected through the hospital data management system, BESTCare 2.0, as well as paper file records and telephone interviews. The pre- and postoperative clinical outcomes were assessed using the Saudi Arabian version of the Knee Injury and Osteoarthritis Outcome Score (KOOS), which is a validated and reliable questionnaire that is used to evaluate the course of knee injury and treatment outcomes [22]. 

The KOOS questionnaire is widely used to assess patients’ perspectives regarding the condition of their knees and includes questions for both sports and daily living activities [23]. It consists of 42 questions about either severity or frequency and includes five subscales: pain (9 questions), symptoms (seven questions), sports and recreational function (five questions), activities of daily living (ADL) (17 questions), and knee-related quality of life (QOL) (four questions). Each question has five possible answers ranging from zero (no problem/none) to four (extreme/always). Each subscale’s score is then transformed to a scale of 0-100 scale, with zero indicating the most severe knee problem and 100 representing no knee problem. The patients answered the KOOS questions both preoperatively and at an average of two years postoperatively. All of them responded except for eight patients who refused the interview and 10 who could not be contacted.

Demographic data (age, sex), health data (body mass index [BMI], diagnosis, comorbidities), intraoperative data (complications and operated knee), and postoperative complications were gathered. A picture archiving and communication system (PACS) system was used to assess all radiographic images. The images were standard standing radiographs (anteroposterior and lateral views) and were evaluated by the same specialized musculoskeletal radiologist for loosening, osteolysis, instability, periprosthetic fractures, and radiolucent lines. 

Osteolysis was defined as an expansile lesion with scalloped margins, while lucency was defined as a radiolucent line found in at least one zone [24,25]. The definition of loosening included either a circumferential radiolucent line measuring >2 mm around the prosthesis, change in alignment, or subsidence of the component [24]. Severe anemia was defined according to the World Health Organization’s (WHO’s) definition as a hemoglobin concentration <8.0 mg/dl [26].

Furthermore, the WHO’s BMI classification was used with the following categories: below 18.5 kg/m^2^: underweight; 18.5-24.9 kg/m^2^: normal weight; 25.0-29.9 kg/m^2^: pre-obesity; 30.0-34.9 kg/m^2^: obesity class I; 35.0-39.9 kg/m^2^: obesity class II; and ≥ 40 kg/m^2^: obesity class III [27]. Classes II and III were combined in the results. 

Statistical analysis

Data were analyzed using the Statistical Analysis System (SAS) version 9.4 for Windows (SAS Institute Inc., Cary, NC, USA). Questionnaire scores were calculated according to the definitions described in the questionnaire. Categorical variables are reported as frequencies and percentages, and quantitative variables are summarized as the mean and standard deviation. The associations of categorical variables with categorical output variables were evaluated using the chi-squared and Fisher exact tests, and the average values of continuous variables were assessed across the categorical outcome variables using a one-way analysis of variance and paired t-tests (which were used to compare pre- and post-operative KOOS subscale scores). Finally, a linear regression analysis was done to assess the independent factors impacting the post-operative KOOS subscale scores (the dependent variables) after adjusting for BMI. A P-value of less than 0.05 was considered statistically significant.

Ethical consideration

Patient confidentiality was maintained throughout the study. Their medical record numbers were de-identified, and access to the data was restricted exclusively to the investigators. 

Verbal consent was obtained from all patients who were contacted for telephone interviews. The interviews were conducted purely by the research team, and verbal consent was found to be more practical as most of the study participants are from rural areas and it is very difficult for them to come to the hospital. The study was approved by the IRB ethics committee of King Abdullah International Medical Research Center (Ref. # RYD-19-419812-111070).

## Results

A total of 46 patients (70 knees) were included. The patients’ mean age was 68 (±9) years, and female patients represented 67% of the sample. Regarding BMI, 14 (30.4%) patients were pre-obese, 11 (23.9%) were class I obese, and 14 (30.4%) were class II. OA was the indication for all surgeries except for one patient who had post-traumatic injury, and 52% had primary bilateral TKA at the same admission (Table 1). The most common comorbidities were hypertension (65.2%), dyslipidemia (52.2%), and diabetes mellitus (50%) (Table 1). No revision cases were reported. Males and patients with bilateral TKA showed significantly higher scores for post-operative sport and recreational activities, as well as QOL (P-value < 0.05). BMI did not have any statistically significant effect on post-operative KOOS subscale scores (Table 2).

**Table 1:** Basic demographics.

Variable	
Age (in years), mean ± SD	68 ± 8.9
Sex, N (%)	
Male	17 (37)
Female	29 (63)
BMI, N (%)	
Pre-obesity	14 (30.4)
Obesity Class I	11 (23.9)
Obesity Class II	14 (30.4)
Obesity Class III	7 (15.3)
Site of operation, N (%)	
Left	14 (30.4)
Right	8 (17.4)
Bilateral	24 (52.2)
Marital status, N (%)	
Single	3 (6.5)
Married	40 (87)
Widowed	3 (6.5)
Comorbidities	
Hypertension	30 (65.2)
Dyslipidemia	24 (52.2)
Diabetes mellitus	23 (50)
Benign prostatic hyperplasia	6 (13)
Hypothyroidism	3 (6.5)
Depression	3 (6.5)
Rheumatoid arthritis	3 (6.5)
Others	27 (58.7)
Length of hospital stay (in days), mean ± SD	14.6 ± 7.4
Days until ambulation, mean ± SD	4.6 ± 2.8

**Table 2:** Linear regression analysis. *P < 0.005. ADL: activities of daily living; QoL: quality of life; TKA: total knee arthroplasty.

Symptom			Pain		ADL		Sport		QOL	
	Coefficient	P-value	Coefficient	P-value	Coefficient	P-value	Coefficient	P-value	Coefficient	P-value
Age	0.300	0.007*	-0.244	0.372	-0.031	0.933	0.535	0.120	1.127	0.027*
Gender	-4.511	0.382	-0.932	0.855	12.638	0.082	16.157	0.014*	29.499	0.003*
Marital status	-3.559	0.396	-1.043	0.803	13.188	0.027*	-0.195	0.970	1.728	0.819
BMI	0.002	0.997	0.871	0.173	0.169	0.830	0.129	0.839	1.002	0.342
Side of TKA	6.24	0.107	3.556	0.405	11.7	0.027*	12.75	0.004*	13.94	0.047*

Regarding postoperative complications, 11 (23.9%) patients developed one short-term postoperative complication, while one (2.2%) patient developed two short-term postoperative complications. Pulmonary embolism (PE) was detected in three patients (6.5%), and deep vein thrombosis (DVT) was detected in one patient. One patient had soft tissue infection, while another had both DVT and soft tissue infection. Only four patients (8.7%) had long-term complications, which were detected clinically (Table 3).

**Table 3:** Complications. PE: pulmonary embolism; DVT: deep vein thrombosis.

Variable	
Short-term complications, N (%)	
PE	3 (6.5)
DVT	2 (4.4)
Soft tissue infection	2 (4.4
Compartment syndrome	0
Deep tissue infection	0
Others	7 (15.2)
Long-term complications, N (%)	
Periprosthetic fractures	2 (4.4)
Stiffness	1 (2.2)
Knee instability	1 (2.2)
Aseptic loosening	1 (2.2)

Furthermore, radiological complications were found among six patients (13%) in six knees (8.6%), with radiolucent lines found in two patients (4.4%) in one knee each (2.9%), periprosthetic fracture in two patients (4.4%) in one knee each (2.9%), signs of loosening in one patient (2.2%) in one knee (1.4%), and instability/dislocation in one patient (2.2%) in one knee (1.4%). There was no radiological evidence of osteolysis. The KOOS subscale scores for both pre- and postoperative intervals were measured. The subscale scores for symptoms, pain, ADL, and QOL all showed statistically significant changes postoperatively (P-value <0.0001; Figure 1).

## Discussion

In our study, the average hospital stay was consistent with reports in the literature and averaged from five to 30 days [2,28,29]. Furthermore, the average time until ambulation in our study was 4.6 days, which was probably due to the motor-weakness side effect of the epidural protocol used. Some studies allowed patients to ambulate from the first day postoperatively, whereas others reported that all their patients ambulated from the second day postoperatively [2,3,13]. In contrast, many studies do not mention a specific ambulation time and only reported that they encouraged patients to ambulate with the help of a walker according to their tolerance [2,30].

Regarding post-operative complications, the rates of wound infection, PE, and DVT in this study were higher than those in other studies [1,4,6,11]. This could have been due to the multiple comorbidities of our patients in addition to the high BMI and relatively advanced age. All TKA patients had a standard postoperative protocol consisting of early ambulation along with mechanical and chemical thromboembolism prophylaxis, which consisted of pneumatic compression stockings, heparin/enoxaparin, and antibiotic prophylaxis.

As for the long-term complications, the number of cases of aseptic loosening was lower than in another study [5], but this may have been due to the smaller sample size in our study. On the other hand, two studies reported no periprosthetic fracture, stiffness, instability, or aseptic loosening, which could be due to the small sample size in these studies compared to ours [2,3]. A long-term study with an average follow-up of 15.2 years and a sample size of 325 patients (347 knees) stated that only four patients (1%) developed long-term complications [3]. Three of them suffered from persistent anterior knee pain during the first year [4]. 

A prospective cohort study examined 208 patients who underwent Medial-Pivot TKA with a follow-up of seven years. They reported only three revisions, and one of them was due to surgical technique error [1]. We suspect that the higher rate of long-term complications in our study is due to the small sample size and high BMI in our patients. For patients who undergo TKAs, we recommend strong education regarding obesity and its effects on the success of MP-TKAs.

Younger age correlated with improved post-operative symptoms and QOL. Furthermore, male gender and bilateral TKA were associated with higher post-operative scores in both sports and QOL, which could be related to the fact that males can be more active when it comes to sports activities in this particular age group. Furthermore, BMI did not seem to have any significant effect on any post-operative KOOS sub-score, but we believe that a significant effect could be observed with a larger sample size. Our postoperative radiographic results were comparable to those in other studies with smaller percentages [14,25]. The radiolucent line detected was stable and did not require any intervention, and loosening did not require any revision at the final follow-up.

In line with our hypothesis, the KOOS sub-scores showed positive results postoperatively in terms of pain alleviation and increased satisfaction, which was reflected by an increase in QOL. These results are consistent with the literature [29]. The sub-scores for sports and recreational activity did not show a statistically significant improvement, which could be attributed to the sedentary lifestyle of our population, as suggested by the high BMI and advanced age.

Our study has certain limitations, including the small sample size, relatively short follow-up period, and retrospective study design. These factors might have prevented us from observing some statistically significant results, such as the effect of BMI on post-operative KOOS subscale scores. Nevertheless, our study is the first of its kind to shed light on the outcomes of this TKA design in Saudi Arabia, and hopefully, it will aid in conducting future studies on this topic. A larger prospective study with a longer follow-up period is needed to confirm the superiority of this design. However, the results cannot be generalized to other countries due to the differences in demographic data and prevalence of medical comorbidities. 

## Conclusions

MP-TKA has been shown to improve QOL, ADL, and pain levels of patients with end-stage OA, as well as low short- and long-term complications. Both male gender and bilateral TKA were associated with improved post-operative symptoms and QOL KOOS sub-scale scores. Our study is the first in the region to investigate the clinical and radiological postoperative outcomes of this TKA design. However, a larger prospective study with a longer follow-up duration is recommended to develop solid evidence regarding the method’s efficiency and benefits.
